# Adherence to screening appointments in a cervical cancer clinic serving HIV-positive women in Botswana

**DOI:** 10.1186/s12889-019-6638-z

**Published:** 2019-03-18

**Authors:** Francis Barchi, Samantha C. Winter, Faith Mompati Ketshogile, Doreen Ramogola-Masire

**Affiliations:** 1 Edward J. Bloustein School of Planning & Public Policy, Rutgers, The State University of New Jersey, 33 Livingston Avenue, New Brunswick, NJ 08901 USA; 2 Rutgers, The State University of New Jersey, New Brunswick, NJ USA; 3 Tati River Clinics, Francistown, Botswana; 4 University of Botswana Faculty of Medicine, Gaborone, Botswana

**Keywords:** HIV, Cervical cancer, Adherence, Cancer screening, Botswana

## Abstract

**Background:**

The link between human immunodeficiency virus (HIV) and cervical cancer is of particular concern in Botswana, where one in four women at risk for cervical cancer is HIV-positive. In settings where co-occurrence of these diseases is high, adherence to screening appointments is essential to ensure detection and early treatment.

**Methods:**

This study took place in a cervical cancer-screening program in an HIV clinic in Botswana. Data for this analysis came from 1789 patient records and 257 semi-structured surveys about the screening consent process that were completed by a subset of patients.

**Results:**

Forty percent of women kept their scheduled follow-up appointments. Findings suggest that women treated at first visit or referred for additional treatment due to the presence of more advanced disease had more than double the odds of adhering to follow-up appointments compared to women with negative screens. Women who completed the 35-min surveys in the embedded consent study were found to have 3.7 times greater odds of adhering to follow-up appointment schedules than women who did not. Factors such as age, education, income and marital status that have been shown elsewhere to be important predictors of adherence were not found to be significant predictors in this study.

**Conclusions:**

HIV-positive women in Botswana who are symptom free at initial screening may be lost to essential future screening and follow-up care without greater targeted communication regarding cervical cancer and the importance of regular screening. Strategies to reinforce health messages using cell phone reminders, appointment prompts at time of anti-retroviral drug (ARV) refills, and use of trained community workers to review cervical cancer risks may be effective tools in reducing the burden of cervical cancer disease in HIV-positive women in this setting.

**Electronic supplementary material:**

The online version of this article (10.1186/s12889-019-6638-z) contains supplementary material, which is available to authorized users.

## Background

Cervical cancer is the 4th most common cause of cancer in women, accounting for 7.9% of all female cancers worldwide [1]. While cervical cancer has become a largely preventable disease in most developed countries due to screening, early detection, and treatment, millions of women worldwide still lack information about the disease, including its risk factors, access to screening and early treatment, and oncology services for the treatment of advanced disease [2]. In 2012, 85% of all cervical cancer cases and 87% of all deaths from cervical cancers occurred in low- and middle-income countries (LMIC) [3]. Southern Africa ranked third among regions in cervical cancer deaths in 2012, with an age-standardized rate (ASR) of 17.9 women per 100,000 compared to a worldwide ASR of 6.8 per 100,000. Cervical cancer is the leading cause of female cancer in Botswana [4]. Despite the government’s strong commitment to primary prevention of cervical cancer through a human papillomavirus (HPV) vaccination program initiated in 2015 and to secondary prevention through an expanding national screening program, the disease remains the country’s leading cause of cancer deaths in women [1].

Among the factors that have been found to increase cervical cancer risk are infection with HPV, long-duration use of hormonal contraceptives by HPV-positive women, smoking, high parity, and co-infection with HIV [5–9]. Women living with HIV are at greater risk for HPV infection and are 2–8 times more likely to develop cervical cancer than uninfected women [9]. Studies have shown that HIV-positive women develop severe pre-cancerous lesions at a younger age than non-infected women (32.7 years versus 47.5 years), experience shorter intervals between onset and invasive disease (3.2 years versus 15 years), and have more recurrences and lower survival rates [10, 11].

Because detection and treatment play a significant role in determining outcomes for women with cervical cancer, considerable attention has been paid by health professionals and researchers to promoting women’s utilization of prevention services. A significant body of research, mainly among women in high-income countries where cytology-based screening is the predominant method of screening, has identified a number of factors associated with non-adherence to screening. They include being older, having less than a high school education, lack of insurance or regular health care, no referral by a health provider, poor acculturation, low income, misconceptions about cervical cancer, and perceived vulnerability to the disease [12–15]. Research focused on HIV-positive women have found that one quarter to one half of this group, while at greater risk of cervical cancer than HIV-negative women, may not have had any screening for the disease [16–19]. Indeed, they were less likely to have had a screening for the disease than their uninfected peers, for whom adherence rates have been reported ranging from 14 to 20% [18]. It has been suggested that preventive care in general may become ‘lost’ in the face of more urgent care for HIV; women may be overwhelmed with too many appointments, often in an environment in which centralized care is absent [17].

Information on factors associated with women’s utilization of cancer prevention services in LMIC countries is far more limited. A 2012 study in a rural district in Tanzania found that distance to a screening facility and women’s knowledge about cervical cancer and its prevention were significantly associated with uptake of screening services [20]. Similarly, time to screening facility was also a factor in a study of 15 countries in sub-Saharan Africa and South Asia [21]. Additional factors, including being younger than 60 years, having a secondary education or higher, and being single were also significantly associated with the likelihood that a woman would have been screened for cervical cancer. Care-seeking from traditional healers and being from rural areas was associated with women not having ever been screened. A 2011 study of 514 women in Zimbabwe reported that women who were financially independent from their husbands and women living in villages were more likely to access cervical cancer prevention services than women who were dependent on their husbands for support or who lived in more remote settings [22]. Age and lack of knowledge about cervical cancer and its prevention as factors associated with screening in Nigeria and Kenya, respectively [23, 24]. In Botswana, two studies that looked at cancer awareness and cancer screenings identified age, lack of knowledge, provider attitudes, and limited access to doctors as factors in women not seeking timely screening for cervical cancer [25, 26]. Literature to date on screening programs targeting HIV-positive women in developing countries has largely concentrated on issues relating to feasibility and scalability; there has been virtually no scholarly work looking specifically at correlates of adherence to cervical cancer screening guidelines among HIV-positive women in Africa and elsewhere in low-resource settings. One 2010 study by Dal Maso and colleagues of screening adherence among women in Northern Italy noted that women born in Africa (39%) were more likely than native-born women not to have had a Pap smear within the past year as recommended [27].

The link between HIV and cervical cancer is of particular concern in Botswana, where two-thirds of cervical cancer patients are HIV-positive [28] and where it is estimated that as many as 200,000 women, i.e., 25% of women at potential risk of cervical cancer, were living with HIV in 2016 [29]. HIV disproportionately affects women in Botswana, with women numbering more than half of all people living with HIV in 2016. Expanding primary and secondary intervention services to reduce the disease burden of cervical cancer in its population has been a major priority of the government of Botswana for more than a decade. The first comprehensive plan for cervical cancer prevention (2012–2016) called for the dual use of visual inspection with acetic acid (VIA) linked to same treatment visit cryotherapy when indicated, as well as Pap smears for coverage of 80% of at-risk women ages 30–49 years [28]. Despite major gains in coverage achieved under the plan, median time from diagnosis to treatment is approximately 4–5 months; the reasons for this are unclear, although distance to treatment center, poor healthcare systems, easier access to traditional healers, opportunity costs, and limited cancer awareness and cancer stigma have been suggested as potential factors. The purpose of this study was to assess the extent to which women who were screened for cervical cancer as part of a pilot program in Botswana kept their follow-on appointments and to identify factors associated with adherence to the prescribed screening schedule at the time of the study.

## Methods

### Setting

This study took place in a cervical cancer-screening program in the Bontleng Clinic in Gaborone, a primary care clinic in the capital city of Botswana. The program was part of a national pilot program undertaken by the Botswana Ministry of Health in cooperation with the Botswana UPenn Partnership and funded by the US President’s Emergency Plan for AIDS Relief (PEPFAR). At the time of this study the Bontleng Clinic was also the site of a district anti-retroviral clinic which drew patients from throughout the city for HIV testing and ARV treatment. Nurses provided brief health educational talks about cervical cancer each morning in the waiting areas of the ARV clinic to encourage women to come for screening. Women interested in taking part could come to be screened during that same visit or return at a later date. As is common in many public healthcare services in Botswana, women were not given specific appointments for their initial screenings but were taken on a first-come, first-serve basis each day at the clinic. Fixed-day appointments were given to women for all follow-on screenings; those women who were treated during their initial visit were to be re-screened at the time of each follow-on appointment.

As part of the national pilot program, cervical cancer screening at the Bontleng Clinic provided an opportunity to examine the acceptability and efficacy of a screening program using Visual Inspection with Acetic Acid (VIA) and Enhanced Digital Imaging (EDI) for the early detection, triage, and treatment of pre-cancerous and cancerous lesions of the cervix [30]. Patients were screened at the clinic by a nurse trained in this “See and Treat” procedure; low-grade lesions were treated with cryotherapy at the same visit. The screening process was enhanced by the use of digital cameras that allowed the nurses to show patients their exam, discuss where a problem was detected and what treatment, if any, was needed. Women with more complex lesions were referred to a specialized clinic at Princess Marina Hospital where a physician would decide on further treatment. The digital images were reviewed on a weekly basis in discussion sessions with the nursing staff and the program’s medical director; the sessions provided opportunities for capacity building as well as program quality assurance. At the time that data were collected for this study, the pilot program was still uncertain as to the extent to which cervical cancer risk was increased by its co-occurrence with HIV and the recommended timing of periodic screening as a prevention tool in HIV-positive women. During the pilot phase, women who screened positive and received same day cryotherapy treatment for pre-cancerous lesions were asked to return for a follow-up screening at six months. Women referred to Princess Marina Hospital for more complex procedures were sometimes asked to return to the clinic in a few weeks for a post-procedure check-up, and women with negative screens were given appointments to return at one year.

### Study samples

**Fig. 1:**
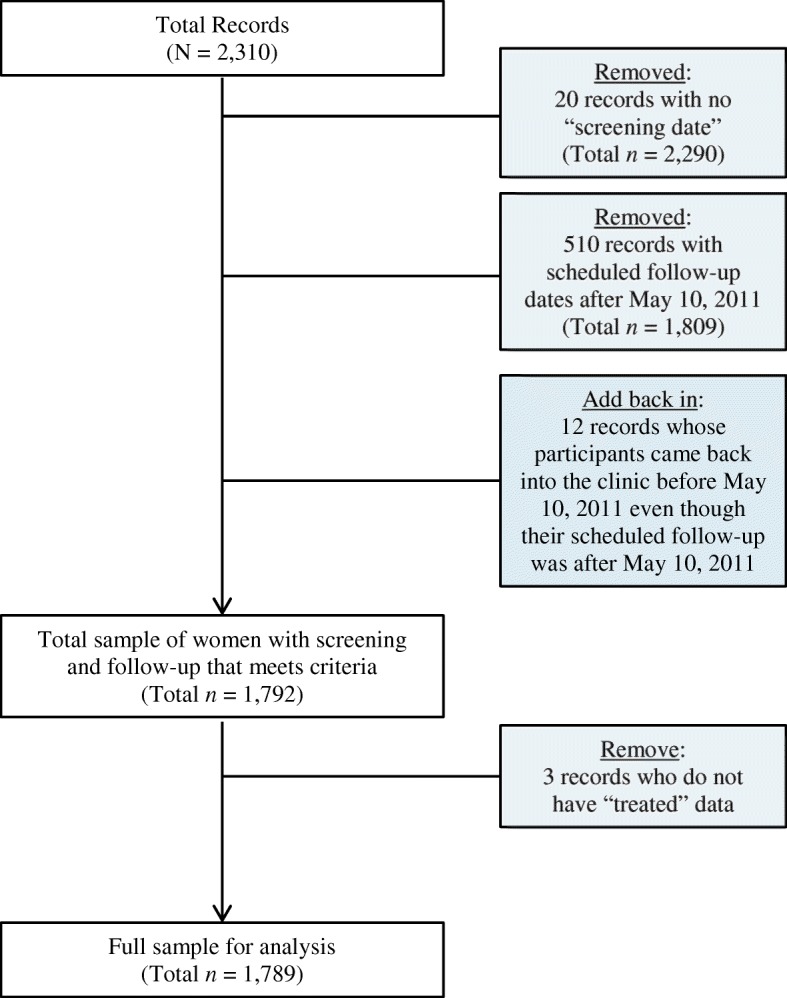
Derivation of full sample (medical records only) for analysis

**Fig. 2:**
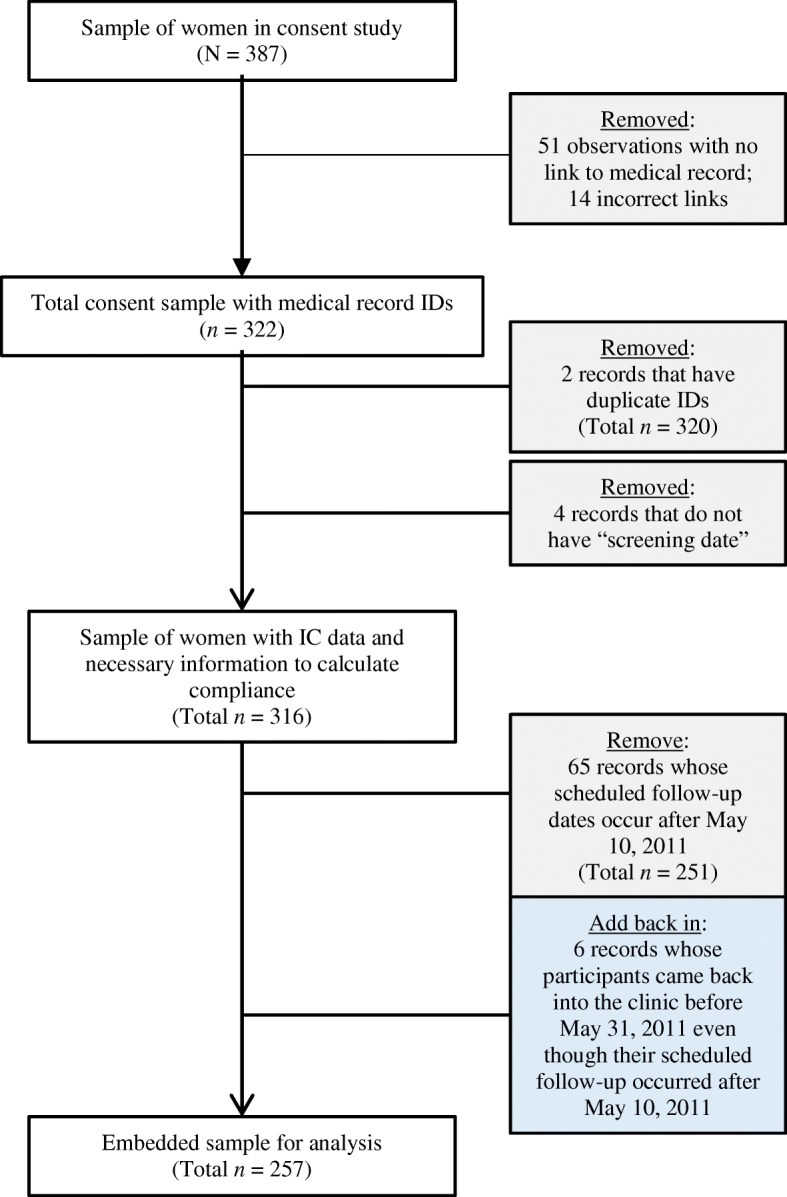
Derivation of embedded sample for analysis

Data for this analysis came from two sources – patient medical records from the screening program dating from its inception in March 2009 through May 10, 2011 (Fig. 1) and an embedded study on the informed consent process that took place from April 2009 – May 10, 2011(Fig. 2). Medical records for 2310 patients were reviewed to identify eligible records, i.e. records containing complete data on dates of first screening and scheduled follow-on appointments, results of initial screening (negative or treatment required) and follow-up appointments scheduled to occur prior to the close of the data collection phase. From the 1789 records that met these criteria, the study team then derived data on women’s screening and treatment schedules as well as basic demographic information on age, education, employment, household income, relationship status, and number of children. All women screened at the clinic were over the age of 18 years.

The second set of data was derived from an embedded study of the informed consent process used as part of patient intake for the VIA screening program at the clinic during an eleven month period in 2010–2011. The purpose of the embedded study was to determine the extent to which participants in the screening program understood the nature and purposes of the screening process and appreciated the risk factors associated with cervical cancer given their HIV status. As part of the embedded study, a member of the study team was present in the clinic on a daily basis during patient intake; the team member approached all women in the waiting area who had completed a patient-intake process with the clinic nurse and provided consent for the screening. Of the 398 women who consented to the screening during the period April 1, 2009 – May 10, 2011, 387 agreed to be part of the embedded consent study. At the request of the clinic staff, no incentives were offered to women for their participation in the embedded study to avoid any possible misperceptions that women who came to the clinic for screening would be paid to do so. The team member explained the purpose of the embedded study and those who agreed to participate were asked to sign or have their oral consent witnessed on a written consent document. Women who agreed to participate were interviewed by the study team individually in a separate office adjacent to the waiting area prior to, or immediately after their exam. A semi-structured interview, conducted in Setswana (the local language in Botswana) and English, was developed specifically for this study to assess women’s knowledge and understanding, based on information they were provided during the consent process, about cervical cancer, the roles of HPV and HIV in the disease, screening-related risks, and the importance of regular preventive care (see Additional file 1). The consent study also gathered demographic information and collected data about women’s general health status, and their roles as decision makers in their daily lives. Of the 387 women who agreed to participate in the embedded study, only 257 could be linked by identifiers (IDs) to medical records and had at least one scheduled follow-up appointment prior to the end of the study period on May 10, 2011. Once a number was assigned to link the medical records of these women to their embedded study questionnaires, data from both sources was entered into documents for this analysis in de-identified form and the linking IDs destroyed.

### Measures

#### Measures from patient records

The outcome variable of interest in this study represents women’s adherence to scheduled appointments. Women who had a negative screen were given appointments to return in 12 months; women who had been treated during the screening visit were, for the most part, given an appointment to return at six months. Finally, in a few cases, women who had been treated required a follow-up appointment within several weeks of their visit. First, a binary variable “complied with instructions to come to the clinic for a follow-up appointment” was created. Women who kept a scheduled follow-up appointment were given a score of 1= “adhered to follow-up schedule”. Women who were assigned a follow-up appointment, but who did not return to the clinic within a 30-day window were given a score of 0= “did not adhere to follow-up schedule”.

Demographic information derived from the patient records included age, education, type of employment, monthly household income, relationship status, and number of children. In cases where women were unaware of their monthly household incomes, imputation was used to provide estimated values for household income based on the sample distribution; however, an additional binary economic variable, ‘aware/unaware of household finances’, was also included in the regression models to account for these women’s responses. An additional variable was created from the logged appointment schedules to indicate whether or not a woman had been treated during any of her appointments at the clinic.

#### Measures from embedded consent study

The information provided by women in the embedded study on informed consent was much more detailed than that collected by the clinic nurse as part of regular patient intake. Measures were created from women’s responses to interview questions about their roles as health decision-makers in general as well as specific questions regarding their understanding of cervical cancer, cervical cancer risk, and the screening process. Binary variables relating to a woman’s health-related decision-making were created from responses to questions about who, in the household, made decisions about having a child, using birth control, buying one’s own medicine, and purchasing other household items such as toiletries. A nine-item depression subset of the full Patient Health Questionnaire (PHQ-9) that had been previously validated in Setswana was used to assess the presence of depressive symptoms in respondents [31, 32]. All women in the embedded sample were asked four questions to ascertain whether or not they had understood the information provided during patient intake; these included questions asking a woman to explain what HPV was, its relation to HIV, the causes of cervical cancer, and the effects of one’s HIV status on the risk of cervical cancer. For these analyses, women who answered all four questions correctly were given a score of 1 = ‘understands risk’ and those who could not answer all four questions correctly were given a score of 0= ‘does not understand risk’. Additional binary variables were created to indicate whether a woman had to consult with or ask permission from someone else prior to taking part in the screening program, and whether she was a returning patient to the screening program. Women’s responses to questions about the nature of their work for wages in the embedded study were more specific than the information gathered during patient intake. Based on responses to interview questions about the actual type of work performed, the study team member was able to be determine whether to classify the work as ‘informal work for wages’ (yes/no) or ‘formal work for wages’ (yes/no) and avoid possible misattribution by respondents.

### Analysis strategy

In order to determine the extent to which women at the Bontleng Clinic adhered to then-current protocols for screening appointments, this analysis examined data from the patient records as well as demographic data and measures of knowledge about cervical cancer from the embedded study. Detailed data from the embedded study on women’s understanding of information provided to them as part of the patient consent process were not included in this analysis and will be published elsewhere when analysis has been completed. Descriptive statistics were run for all variables of interest for the full and embedded study sample separately. Bivariate analyses were used to examine associations between women’s adherence and each of the variables of interest (Pearson’s chi-squares for Fisher’s Exact Tests, depending on bin size, for binary and categorical variables of interest and logistic regressions for continuous variables of interest). Two separate logistic regression models were run in Stata statistical software version 14 to explore associations between a number of factors and women’s medical adherence [33]. The first model only included data derived from medical records. The second model looked at data from the survey responses of women who participated in the embedded study combined with the relevant medical record information. The proportion of missing values on all variables of interest was 5% or less; thus, a user-written program (hotdeckvar) in Stata v.14 was used to carry out single value imputation on all of the variables used in the models except the treated variable and the outcome adherence variable [34].

## Results

### Descriptive statistics

Descriptive statistics are summarized in Table 1. Findings show that many of the descriptive statistics are similar between the full and embedded samples—suggesting the embedded sample is probably a good representation of the full sample. Of the 1789 women whose medical records indicated return screenings/check-ups scheduled during the data collection period, 718 women were adherent to within 30 days of their appointments. About 10% of the women in the full sample (*n* = 176) were screen-positive and required treatment for pre-cancerous lesions during their clinic visit or were referred to Princess Marina Hospital for treatment for cancerous lesions. Of those women, 109 (about 62%) were adherent to within 30 days of their appointments. By comparison, of the 1789 women, 1612 were screen-negative (not treated), and of those women, 609 (34%) were adherent to within 30 days of their appointments.

**Table 1:** Descriptive Statistics

	Full Sample				Embedded Sample			
	Frequency	Did not comply	Complied	Χ^2^	Frequency	Did not comply	Complied	Χ^2^
*N*	1789	1071	718		257	83	174	
Participated in consent study	257	83	174 (68%)	94.94***	NA	NA	NA	
Did not participate	1532	988	644 (42%)					
Demographic Variables								
Age	34.9 (7.01)			0.02*†	35.2 (6.42)			0.03†
Education				1.05				0.89
No formal education	52	31	21		62	17	45	
Primary	428	250	178					
Secondary	1220	733	487		195	66	129	
Higher	89	57	32					
Employment type				11.26*	NA	NA	NA	NA
Unemployed	572	368	204					
Formal sector	950	535	415					
Informal sector	177	114	63					
Self-employed	90	54	36					
Income				3.75				8.43*
Less than p1000	806	463	343		108	29	79	
p1000-p2499	688	425	263		103	38	65	
p2500-p4999	204	125	79		28	6	22	
p5000-p10000	91	58	33		18	10	8	
Aware of household finances	1228	704	524	10.49**	192	57	135	2.36
Relationship status				3.70				1.42†
Single	1201	712	489		186	58	128	
Cohabitates	266	163	103		23	7	16	
Married	235	150	85		37	15	22	
Separated/div/widow	87	46	41		11	3	8	
Number of children	2.1 (1.39)			0.03†	2.0 (1.29)			0.11†
Treated	176	67	109	38.60***	40	4	36	10.77†**
Additional variables for embedded sample								
Formally employed					180	56	124	0.39
Engages in informal work					67	14	53	5.39*
Involved in decisions about family planning					156	50	106	0.01
Involved in decisions about buying medicine					241	76	165	1.02
Involved in decisions about other household items					240	77	163	0.07
Symptoms of major depressive disorder					16	6	10	0.21
Correctly identified causes of cervical cancer					102	37	65	1.22
Understands role of HIV in cancer risk					245	79	166	0.01†
Identified HPV as a cause of cervical cancer					130	43	87	0.07
Understands role of HIV in risk of HPV					148	49	99	0.11
Composite risk variable					75	27	48	0.66
Consulted with someone to participate					169	59	110	1.54
Needed permission to participate					7	4	3	2.03†
Type					126	21	105	27.61***

† coefficient from a logistic regression or Fisher’s Exact Test; *p < 0.05, **p < 0.01, ***p < 0.001

Sixty-eight percent of the women in the embedded sample, i.e., those women who participated in the separate consent study, met the study criteria for adherence, compared to 36% of the women who did not. Of the 257 women in the informed consent sample, 67 were screen-positive and treated and of those women, 53 (79%) were adherent to within 30 days of their appointments. Of the 257 women, 217 were screen-negative (not treated), and of those women, 138 (64%) where adherent.

The average age of women in both the full and embedded samples was approximately 35 years. About 70% of women in the study were single with close to 14% of the remaining women reporting that they were legally married. Approximately one-quarter of the women in the full and embedded samples had a primary education or less and the remaining women had at least a secondary education. Over 40% of both samples had monthly incomes of less than 1000 Botswana pula (approximately $100 US) and an additional 40% had monthly incomes between 1000 and 2499 pula ($100 - $250 US). Around 30% of women, however, did not know their household income, suggesting that they had little control over or access to a predictable monthly income on which to live. Within the embedded sample, about 94% of the women reported that they were involved in decisions about buying toiletries and small household goods and 62% reported being involved in decisions about family planning. Six percent of women had symptoms of depression as defined by the PHQ-9. About 29% of them understood the risks associated with HPV, HIV, and cervical cancer. Two in three women reported that they consulted with someone prior to participating in the study and 2.7% reported needing permission to participate in the study.

In the full sample, factors of significance at the bivariate level of analysis included education, type of employment, awareness of household finances, and treatment at first screening. In the embedded study (which included additional variables about the patients) factors that were significantly correlated with adherence included participation in the embedded consent study, age, income, treatment at first screening, informal sector work, and new/returning patient status at the screening program.

### Factors associated with adherence

Results from the logistic regression of women’s adherence on a variety of factors in the full and embedded samples are summarized in Table 2. According to results, women who participated in the embedded consent study had much higher odds of complying with instructions to return for a follow-up visit (OR 3.7, CI95% 2.79,4.97, *P* < 0.001) than women who did not. Results also suggest women who were treated for pre-cancerous lesions or early symptoms of disease after their initial screening had significantly higher odds of complying with instructions to return for a follow-up visit compared to women who were not treated. The odds of adherence more than doubled for women who were treated in the full sample (OR 2.5, CI95% 1.92,3.55, *p* < .001) and increased by more than nine-fold for women who were treated in the embedded sample (OR 9.11, CI95% 2.72,30.49, p < .001). Only one additional factor emerged as being significantly associated with adherence in the full sample—awareness of household finances. Women who were aware of their household finances in the full sample had 69% greater odds of complying with instructions to return for a follow-up visit than women who did not known their household’s monthly income. This effect, however, did not emerge as a significant factor in the embedded sample that controlled for additional attributes.

**Table 2:** Models

	Full Sample (*n = 1789*)			Embedded Sample (*n = 257*)		
	Odds Ratio	*p*-value	CI (95%)	Odds Ratio	*p*-value	CI (95%)
Included in embedded sample	3.73	0.000	2.792–4.971	–	–	–
Demographic variables						
Age	1.01	0.106	0.997–1.031	1.02	0.615	0.955–1.08
Education (ref: none)						
Primary	0.96	0.883	0.518–1.762			
Secondary	0.98	0.938	0.531–1.796	1.29	0.559	0.549–3.036
Higher	0.93	0.852	0.431–2.007			
Employment type (ref: unemployed)						
Formal sector	0.79	0.384	0.457–1.352	–	–	–
Informal sector	0.57	0.075	0.307–1.058	–	–	–
Self-employed	0.80	0.472	0.43–1.479	–	–	–
Income (ref: more than p5000)						
Less than p1000	1.58	0.075	0.955–2.615	3.46	0.056	0.967–12.367
p1000-p2499	1.22	0.431	0.741–2.022	2.14	0.223	0.628–7.311
p2500-p4999	1.27	0.390	0.733–2.214	3.14	0.133	0.706–13.951
Aware of household finances	1.73	0.046	1.009–2.956	1.10	0.803	0.528–2.278
Relationship status (ref: single)						
Cohabitates	0.98	0.907	0.738–1.309	0.57	0.342	0.181–1.809
Married	0.80	0.168	0.588–1.097	0.72	0.487	0.283–1.825
Separated/div/widow	1.25	0.347	0.784–1.999	2.61	0.252	0.506–13.476
Number of children	1.01	0.806	0.932–1.094	1.18	0.254	0.887–1.573
Treated	2.54	0.000	1.821–3.548	9.11	0.000	2.721–30.492
Additional variables for embedded sample						
Formally employed				1.42	0.392	0.635–3.194
Engages in informal work				2.30	0.053	0.99–5.349
Involved in decisions about family planning				0.99	0.984	0.519–1.901
Involved in decisions about buying medicine				4.90	0.078	0.835–28.708
Involved in decisions about health care				0.30	0.214	0.045–1.999
Symptoms of major depressive disorder				0.85	0.796	0.240–2.987
Composite risk variable				0.59	0.136	0.294–1.180
Consulted with someone to participate				0.55	0.097	0.274–1.113
Needed permission to participate				0.38	0.286	0.065–2.241
Type				5.65	0.000	2.883–11.080
*N*	*1789*			*257*		

In the embedded sample, two additional factors emerged as being significantly associated with women’s adherence—their participation in informal work and if they were a returning patient to the clinic. Women who reported having informal work at the time of the study had over twice the odds of adhering compared to other women in the study. Women who reported that they had been patients of the clinic prior to the study had over five times the odds of complying with instructions to return for a follow-up visit after their screening compared to women who, at the time of the study, were new patients to the clinic (OR5.65, CI95% 2.88, 100.98, *p* < .001).

## Discussion

Adherence to appointments for cervical cancer screening and treatment is important for women everywhere but is critically so in countries in which a high percentage of the female population is HIV-positive and therefore at increased risk of the disease. In such environments, prevention efforts must not only encourage screening for all women in accordance with WHO guidelines [36] but must also aggressively target HIV-positive women in whom there is currently no evidence of the disease but for whom the risks of developing the disease remains high. This is the first empirical study in Botswana to examine the factors that may influence whether or not a woman adheres to instructions that she receives with respect to follow-up screening appointments and to consider the implications for the success of screening programs in reducing the incidence and mortality from this disease.

This study provides strong evidence, both at the bivariate and multivariate level of analysis, of a correlation between women’s adherence to appointment schedules and having received treatment or a referral for treatment at their screening appointments. Women who were treated during or as a result of a screening visit were significantly more likely to keep subsequent appointments than women who had negative screens. There may be a number of explanations for this result. Socio-behavioral models of compliance with medical regimens have identified a reliable relationship between compliance and a patient’s subjective perception of the illness threat, the likelihood of susceptibility, the severity of the illness, and the benefits and costs associated with adherence to a recommended medical regimen [35–39]. In his conceptualization of the Health Belief Model, Becker postulated that the presence of physical symptoms may introduce or elevate a sense of reality about the presence and severity of a disease or disease threat that motivates patients to follow medical instruction [36]. Women in this study who were diagnosed with pre-cancerous or cancerous lesions and were treated during their initial screening have experienced physical symptoms associated with that treatment which can reinforce the perception of illness threat. This perception may be further reinforced by the use of EDI cameras as part of the VIA screening process; women with lesions or other abnormalities of the cervix could actually ‘see’ the signs of disease made visible by acetic acid. A visual image of abnormality may drive home the reality of disease threat for treated women; women who at first screening received no such visual reinforcement may perceive their risk for cervical cancer (what Becker terms ‘susceptibility’) to be low. Under such circumstances the ‘costs’ of adherence to future appointments may be deemed too high for the anticipated potential ‘benefits’ to be derived from continued surveillance. This may be particularly true in this sample of women, for whom the perceived threat and severity of AIDS is a daily reality and in a setting where the cost-benefit calculus for adherence to anti-retroviral therapy is continually being reinforced through public campaigns.

A number of intrinsic and extrinsic factors have been shown in previous studies to influence women’s accessing cervical cancer screening, including knowledge about cervical cancer [20, 21, 23–25], distance to screening center [20, 21], age [21, 23, 24], education [21], income/financial independence [21, 22, 40], and marital status [21]. Distance and travel time to clinic, factors previously shown to be significant barriers or enablers of utilization of cancer screening services, were not included in this study, given that the Bontleng Clinic serves a predominantly local constituency. Although age was a significant correlate of adherence at the bivariate level in both the full and embedded samples, it lost its significance in both samples when additional explanatory factors were taken into account. Education and knowledge about cervical cancer (including causes, risk factors, and relationship to HIV-status) were not found to be significant. Similarly, marital status was not significant at any level of analysis. Findings from other studies have suggested that male partners exert significant influence on whether or not a woman accesses cancer screening services [40]; while women in the embedded sample often felt the need to consult with others about taking part in the screening program, fewer than 5% reported needing permission and neither need was a significant factor in the multivariate models. Various dimensions of women’s knowledge of, access to, and sources of income were important in this study. In the full sample, while employment and household income were not shown to be significant in the regression model, being aware of one’s household finances increased the odds that a woman would adhere to screening appointments by almost 70%. While knowing household income does not imply either access to or control over resources, ‘awareness’ does suggest that women are, at least to some extent, involved in the management of these resources; such women may have greater latitude in setting aside household responsibilities to attend to their healthcare needs than women with no awareness of, and, perhaps by extension, only limited access to financial resources. In the regression model for the embedded study, the ‘aware of household finances’ variable lost its significance but, interestingly, ‘engages in informal work’ was found to more than double the odds of a woman’s adherence to screening appointments. One possible explanation for this finding may be that women who work in the informal economy in Botswana do so as self-employed vendors of cell phone time, fruit and candy, and/or handicrafts or pick up work as day-laborers hired under government relief schemes. These types of work may give women greater flexibility in their schedules than women who have steady jobs or who are engaged full-time as subsistence householders, and therefore make it easier for them to make their scheduled follow-up appointments.

At the time of this study, the cervical cancer screening program at the Bontleng Clinic was providing information critical to decision-making by the Botswana government about the feasibility and scalability of a ‘see and treat’ approach to cervical cancer prevention and care. As such, it was important that the program resemble as closely as possible the approach to patient intake and appointment follow-up that would be feasible were the program to be adopted by the Botswana Ministry of Health as a regular part of national health care delivery. Data on patient intake forms were similar to that collected elsewhere in the health care system. No follow-up strategies were used to ensure that women adhered to appointment schedules given that such efforts would not have been scalable given human and financial resources at the time in Botswana’s health system. Instead, it was anticipated that rates of adherence would increase by situating cervical cancer screening and care within the HIV clinic, where new and returning patients to the program (including those requiring post treatment attention following LEEP/colposcopy procedures at the tertiary hospital) already came on a monthly basis for refills of their ARV medications from the clinic dispensary.

Findings from this study suggest a number of possible avenues that the Botswana government might adopt in the future to improve women’s adherence to screening programs. Of particular interest is the finding that women who participated in the embedded consent study had significantly greater odds of adhering to future screening appointment schedules than women who had not. Although the consent study was intended to gather, rather than impart, information, it may well have seemed to patients that staff at the clinic were taking a genuine interest in them as individuals. Time invested by study personnel in establishing personal rapport with women may help establish trust and feelings of social support, factors previously identified as being positively associated with the likelihood of women attending and being treated in cervical cancer screening programs [40]. Another possible explanation for the significant difference in adherence rates between those women who participated in the consent study (68%) and those who did not (42%) may be found in the work to date on improving patient recall of health care information both in research and clinical settings [41, 42]. Key messages imparted during patient intake and the consent process for the screening may have been reinforced when women were asked in the interviews to recall what they had been told about HPV, HIV, and cervical cancer. Although comparison data for the women who did not participate in the consent study is unavailable, the number of women in the consent study who could, for example, remember the role of HIV in cervical cancer risk was very high (95%) and may well have contributed to their attention to follow-on appointments. Although time is a precious commodity in health care settings in general, and particularly in resource-constrained clinics, the use of trained students and community members as in-house health communicators may enhance women’s clinical experiences and their adherence to follow-on appointments without creating additional burden for nurses and physicians.

The use of visual aids in the screening process may be important in how women perceive the severity and threat of cervical cancer, particularly among women in whom pre-cancerous lesions are found. Absent screen-negative findings, HIV-positive women may not grasp the extent to which they remain at risk for cervical cancer and the ongoing need for periodic screening. Strategies to reinforce women’s appreciation of their susceptibility to cervical cancer should be pursued as part of an expanding national screening program in Botswana. Use of cell phone appointment reminders and the inclusion of screening prompts for women when they visit their ARV clinics each month for pharmacy refills may be effective tools in this regard. Health promotion efforts specifically addressing the positive effects of early detection and routine screening for cervical cancer may increase women’s perception of benefit and reduce what may be seen as the opportunity costs involved in adhering to yet another set of medical protocols for women living with HIV.

This study had several limitations that may impact its findings and their interpretation. The data for this analysis were cross-sectional, so no definitive conclusions can be drawn about the temporal or causal relationship among the study variables. In addition, while the data do provide important information on women’s adherence to their appointments, it did not permit us to distinguish with certainty between those women who received same day treatment with cryotherapy for pre-cancerous lesions and those who were referred to the tertiary hospital for more complex procedures. Importantly, this study was undertaken during a pilot phase of the VIA screening program in Botswana at a time when the risk relationship between HIV and cervical cancer was less well understood, and the most effective mode of delivery for a cervical cancer screening program not yet identified. More research on adherence is needed now that a formal national cervical screening and treatment program is in place in Botswana and new policies have been issued with respect to the recommended frequency and types of screening. In addition, the Botswana Ministry of Health implemented in 2015 a national HPV vaccination program as standard of care for girls ages 9–13 years, which is likely to raise awareness in the country about cervical cancer and may significantly impact the number of women who adhere to national guidelines regarding routine screening for the disease [43].

## Conclusions

Expanding primary and secondary intervention services to reduce the disease burden of cervical cancer in its population has been a major priority of the government of Botswana and its international partners for more than a decade. Despite gains in coverage, more work is needed to improve adherence among HIV-positive women to screening appointment recommendations and to achieve the government’s recommended target of 80% coverage of women ages 30–49 years. Given the on-going risk of cervical cancer and its recurrence among HIV-positive women, efforts at raising awareness of disease threat in this population and the deployment of strategies to encourage adherence should be aggressively pursued. HIV-positive women in Botswana who are lesion free at a first screening may be lost to essential future treatment and follow-up care without greater targeted education regarding cervical cancer risks and the importance of regular screening in this population.

## Additional file

Additional file 1: Informed Consent Study Interview Instrument. (PDF 122 kb)

## Abbreviations

ARV: Anti-retroviral drug
ASR: Age-standardized rate
EDI: Enhanced digital imaging
HIV: Human immunodeficiency virus
HPV: Human papillomavirus
ID: Identifier
LMIC: Low- and middle-income country
PEPFAR: US President’s Emergency Plan for AIDS Relief
PHQ-9: Patient Health Questionnaire 9
VIA: Visual inspection with acetic acid

## References

[1] Ferlay J, Soerjomataram I, Ervik M (2013). GLOBOCAN 2012 v1.2, Cancer incidence and mortality worldwide: IARC CancerBase no. 11 [internet].

[2] Chidyaonga-Maseko F, Chirwa ML, Muula AS. Underutilization of cervical cancer prevention services in low and middle income countries: a review of contributing factors. Pan Afr Med J. 2015;21(1). 10.11604/pamj.2015.21.231.6350. 10.11604/pamj.2015.21.231.6350 PMC4607967 26523173

[3] GLOBOCAN (2013). 2012 v1.2, Cancer incidence and mortality worldwide: IARC CancerBase no. 11 [internet].

[4] Bruni L, Barrionuevo-Rosas L, Albero G, et al. ICO information Centre on HPV and Cancer (HPV information Centre). Human papillomavirus and related disease in Botswana. Summary Report 27 July 2017. https://hpvcentre.net/statistics/reports/BWA.pdf. Accessed 1 Feb 2018.

[5] Waggoner SE (2003). Cervical cancer. Lancet..

[6] Smith JS, Green J, De Gonzalez AB (2003). Cervical cancer and use of hormonal contraceptives: a systematic review. Lancet.

[7] International Collaboration of Epidemiological Studies of Cervical Cancer (2007). Cervical cancer and hormonal contraceptives: collaborative reanalysis of individual data for 16 573 women with cervical cancer and 35 509 women without cervical cancer from 24 epidemiological studies. Lancet..

[8] Kjellberg L, Hallmans G, Åhren AM (2000). Smoking, diet, pregnancy and oral contraceptive use as risk factors for cervical intra-epithelial neoplasia in relation to human papillomavirus infection. Br J Canc.

[9] Abraham AG, D’Souza G, Jing Y (2013). Invasive cervical cancer risk among HIV-infected women: a north American multicohort collaboration prospective study. J Acquir Immune Defic Syndr.

[10] Danso D, Lyons F, Bradbeer C (2006). Cervical screening and management of cervical intraepithelial neoplasia in HIV-positive women. Int J STD AIDS.

[11] Fruchter RG, Maiman M, Sedlis A (1996). Multiple recurrences of cervical intraepithelial neoplasia in women with the human immunodeficiency virus. Obstet Gynecol.

[12] Ackerson K, Gretebeck K (2007). Factors influencing cancer screening practices of underserved women. J Am Assn Nurse Pract.

[13] Heberer MA, Komenaka IK, Nodora JN (2016). Factors associated with cervical cancer screening in a safety net population. World J Clin Oncol.

[14] Limmer K, LoBiondo-Wood G, Dains J (2014). Predictors of cervical cancer screening adherence in the United States: a systematic review. J Adv Pract AIDS Care.

[15] Martín-López R, Hernández-Barrera V, De Andres AL (2010). Breast and cervical cancer screening in Spain and predictors of adherence. Eur J Canc Prev.

[16] Baranoski AS, Horsburgh CR, Cupples LA, Aschengrau A, Stier EA (2011). (2011). Risk factors for nonadherence with pap testing in HIV-infected women. J Women Health.

[17] Lambert CL (2013). Factors influencing cervical cancer screening in women infected with HIV: a review of the literature. J Assoc Nurses AIDS Care.

[18] Oster AM, Sullivan PS, Blair JM (2009). Prevalence of cervical cancer screening of HIV-infected women in the United States. J Acquir Immune Defic Syndr.

[19] Tello MA, Jenckes M, Gaver J (2010). Barriers to recommended gynecologic care in an urban United States HIV clinic. J Women Health.

[20] Lyimo FS, Beran TN (2012). Demographic, knowledge, attitudinal, and accessibility factors associated with uptake of cervical cancer screening among women in a rural district of Tanzania: three public policy implications. BMC Pub Health.

[21] Akinyemiju TF (2012). Socio-economic and health access determinants of breast and cervical cancer screening in low-income countries: analysis of the world health survey. PLoS One.

[22] Mupepi SC, Sampselle CM, Johnson TR (2011). Knowledge, attitudes, and demographic factors influencing cervical cancer screening behavior of Zimbabwean women. J Women Health..

[23] Nwankwo KC, Aniebue UU, Aguwa EN, Anarado AN, Agunwah E (2011). Knowledge attitudes and practices of cervical cancer screening among urban and rural Nigerian women: a call for education and mass screening. Eur J Canc Care.

[24] Were E, Nyaberi Z, Buziba N. Perceptions of risk and barriers to cervical cancer screening at Moi teaching and referral hospital (MTRH), Eldoret, Kenya. Afr Health Sci. 2011;11(1). PMC3092325 21572858

[25] McFarland DM (2003). Cervical cancer and pap smear screening in Botswana: knowledge and perceptions. Int Nurs Rev.

[26] Mingo AM, Panozzo CA, DiAngi YT (2012). Cervical cancer awareness and screening in Botswana. Int J Gynecol Canc.

[27] Dal Maso L, Franceschi S, Lise M, de'Bianchi PS, Polesel J, Ghinelli F, Falcini F, Finarelli AC (2010). Self-reported history of pap-smear in HIV-positive women in northern Italy: a cross-sectional study. BMC Cancer.

[28] Grover S, Raesima M, Bvochora-Nsingo M, et al. Cervical cancer in Botswana: current state and future steps for screening and treatment programs. Frontiers Oncol. 2015;5(239). 10.3389/fonc.2015.00239. 10.3389/fonc.2015.00239 PMC4630577 26579491

[29] Joint United National Program on AIDS (2017). Ending AIDS: Progress towards 90–90 – 90 targets.

[30] Ramogola-Masire D, de Klerk R, Monare B, et al. Cervical cancer prevention in HIV-infected women using the “see and treat” approach in Botswana. J Acquir Immune Defic Syndr. 2012;59(3). 10.1097/QAI.0b013e3182426227. 10.1097/QAI.0b013e3182426227 PMC3884088 22134146

[31] American Psychiatric Association. Diagnostic and statistical manual of mental disorders (DSM-IV-TR). 4th ed. Washington DC; 2000.

[32] Lawler K, Mosepele M, Seloilwe E (2011). Depression among HIV-positive individuals in Botswana: a behavioral surveillance. AIDS Behav.

[33] StataCorp (2015). Statistical software (version 14.0).

[34] Schonlau M (2012). Stata-ado package “hotdeckvar” for single hotdeck imputation.

[35] World Health Organization (2013). WHO guidelines for screening and treatment of precancerous lesions for cervical cancer prevention.

[36] Becker MH (1974). The health belief model and sick role behavior. Health Educ Monogr.

[37] Levanthal H, Diefenbach M, Leventhal EA (1992). Illness cognition: using common sense to understand adherence and affect cognition interactions. Cognit Ther Res.

[38] DiMatteo MR, Haskard KB, Williams SL (2007). Health beliefs, disease severity, and patient adherence: a meta-analysis. Med Care.

[39] Ibekwe CM, Hoque ME, Ntuli-Ngcobo B (2010). Perceived susceptibility of cervical cancer screening among women attending Mahalapye district hospital, Botswana. Southern Afr J Epidemiol Infect.

[40] Bingham A, Bishop A, Coffey P (2003). Factors affecting utilization of cervical cancer prevention services in low-resource countries. Salud Publica Mex.

[41] Simoni JM, Amico KR, Pearson CR, Malow R (2008). Strategies for promoting adherence to antiretroviral therapy: a review of the literature. Curr Infect Dis Rep.

[42] Watson PW, McKinstry B (2009). A systematic review of interventions to improve recall of medical advice in healthcare consultations. J Roy Soc Med.

[43] Raesima MM, Forhan SE, Voetsch AC, Hewitt S, Hariri S, Wang SA, Pelletier AR, Letebele M, Pheto T, Ramogola-Masire D, El-Halabi S (2015). Human papillomavirus vaccination coverage among school girls in a demonstration project-Botswana, 2013. MMWR Morb Mortal Wkly Rep.

