# Supplementary material for: Adherence to screening appointments in a cervical cancer clinic serving HIV-positive women in Botswana
Source: BMC Public Health. 2019 Mar 18;19:318. doi: 10.1186/s12889-019-6638-z (PMC6423763; doi:10.1186/s12889-019-6638-z)
Supplement: Supplementary file 1 — Informed Consent Study Interview Instrument. (PDF 122 kb) [file 12889_2019_6638_MOESM1_ESM.pdf]

Date: _________________
Start time: _____AM/PM
End time: _____AM/PM

Study Interview Instrument

You have just been invited to participate in a screening program for cervical cancer here at the Bontleng Clinic. I would like to ask you a few questions about the screening program as well as some questions that will help us know more about women in Botswana and how they make decisions about people and situations in their daily life. This information will help us understand how we may improve the way in which we explain research studies to other women like yourself in the future. **O sa tswa go lalediwa go tsenelela lenaneo la tlhatlhobo ya kankere ya molomo wa popelo mo tliliniking ya Bontleng mo. Ke eletsa go go botsa dipotso di se kae mabapi le lenaneo le, le dipotso tse dingwe tse di ka dirang gore re itse thata ka basadi ba Botswana le ka fa ba fetsang megopolo ka batho ba bangwe le ka diemo dingwe mo matshelong a bone a malatsi otlhe. Kitso e e ka re thusa go lemoga gore re ka tla ra tlhabolola jang tlhaloso ya dipatlisiso tsa mofuta o mo basading ba bangwe jaaka wena.**

Please remember that if you do not want to answer any specific questions, you can let me know and I will go on to the next question. Also, please let me know if you would like me to repeat any questions, if you don't understand a question, or if you would like more time to think about a question. You may stop this interview at any time. Whether you answer a question or not will not affect the care you receive in this clinic nor whether or not you may participate in the cervica cancer screening program. **Tswee tswee, gakologelwa gore fa o sa batle go araba dipotso dingwe o ka nkisitse mme ka fetela kwa go tse dingwe. Gape, nkitsise fa o batla ke boelela dipotso dingwe, fa o sa tlhaloganya sentle, kgotsa fa o batla sebakanyana sa go akanyetsa potso. O ka emisa potsolotso e ka nako nngwe le nngwe. Le fa o sa arabe potso nngwe ga go na go kgoreletsa thuso e ka e fiwang mo tliliniking e, le o ka seke o tsenelele tlhatlhobo ya kankere ya popelo tota.**

Please answer these questions as honestly and carefully as you can. The information you give me in this interview will be kept confidential, and there will be no way that it can be linked to you. **Tswee tswee, araba dipotso ka boammaaruri jotlhe jo o ka bo kgonang, le ka matsetseleko. Se o se buang mo potsolotsong e e tla nna sephiri, ebile ga go ka fa se ka tlholang se golaganngwa le wena ka teng.**

**Screening participation**	Participation status	YES, AGREED TO PARTICIPATE……	1
		NO, REFUSED TO PARTICIPATE……	0
		INELIGIBLE……………………………	88
	Type of patient	NEW PATIENT………………………..	0
		FOLLOW-UP……………………………	1

Thank you for talking with me. I would like to begin by asking you questions about your daily life, your home and your family. If you do not want to answer any specific questions, you can let me know and I will go on to the next question. Also, please let me know if you would like me to repeat any questions, if you don't understand a question, or if you would like more time to think about a question.
Ke lebogela sebaka se sa go bua le wena. Ke eletsa go simolola ka go go botsa dipotso ka botshelo jwa gago jwa malatsi otlhe, lelwapa la gago, le ba lelwapa la gago. Fa o sa rate go araba dipotso dingwe o ka nkitsise mme ka tswelela ka potso e e latelang. Nkitsese gape fa o batla ke boelela potso fa o sa e tlhaloganya sentle, kgotsa o tlhoka sebakanyana sa go e akanyetsa.

		Questions and Filters	Coding Categories	Code
**Age**	AU1	**In what year were you born?**<br>*O tshotswe leng?*	Year Or Birth …………….. ... .<br>No Response …………………. .. .	____<br>99
**Education**	AU2	**Have you ever attended school?**<br>*A o kile wa tsena sekolo?*	Yes ………………………………<br>No ………………………………..<br>No Response …………….. ……<br><br>[* If 'No' (0), Skip To AU4.]	1<br>0*<br>99
	AU3	**What is the highest level of school you attended: primary, secondary, or higher?**<br>*O eme fa kae kwa sekolong: Sekolo se Sennye; Sekolo se Segolwane; Dithuto tse Dikgolo?*	Primary ……………………........<br>Secondary………………………...<br>Higher ……………………………<br>Highest standard/form/grade completed…………………………<br><br>Not Applicable …………………..<br>No Response……………………..	1<br>2<br>3<br><br>____<br><br>88<br>99
**Current employment**	AU4	**Are you currently working for wages outside the home? [FORMAL EMPLOYMENT]**<br>*A mo nakong eno o a bereka?*	Yes………………………………<br>No………………………………..<br>No Response……………………<br><br>[* If 'No' (0), Skip To AU6.]	1<br>0*<br>99
**Type of employment**	AU5	**What kind of work do you perform for your wages? [FORMAL EMPLOYMENT]**<br>*O bereka tiro ya mofuta mang?*<br><br>[IF SELF-EMPLOYED, CHECK HERE_____]	Specify<br>____________________________<br>Domestic/Agricultural<br>Administrative<br>Managerial<br>Other<br>Retail<br>Education<br><br>Not Applicable……………........<br>No Response…………………….	 <br>1<br>2<br>3<br>4<br>5<br>6<br><br>88<br>99
	AU6	**Are there any activities you do to make extra money? [INFORMAL WORK]**	Yes………………………………..<br>What kind of activities?<br>____________________________<br>____________________________<br>No………………………………...<br>No Response……………………...	 <br><br><br>0<br>99
**Monthly household income**	AU7	**What is the average TOTAL monthly income in your household?**<br>*Go tsena madi a selekanyo se se kae mo lapeng ka kgwedi?*	**If amount specified, enter here** ⟶<br><br>THEN CODE BY CATEGORY:<br>≤ 250 Pula/Month…………..<br>> 250 Pula/Month But ≤400 Pula/Month………………………<br>>400 Pula/Month………………..<br>Doesn't Know…………………..<br>No Response………...…………..	_____<br><br><br>1<br><br>2<br>3<br>4<br>99

		Questions and Filters	Coding Categories	Code
**Ethnic group identity**	AU8	**Which home (village) do you come from?**<br>*O tswa kae kwa gae?*<br><br>**Which tribe do you belong to?**<br>*O motho wa morafe ofe?*<br><br>[Code as "Tswana": Rolong, Ngwato, Lete, Tlokwa, Kwena, Kgatla, Ngwaketse, Tawana]	Tswana……………………………<br>Kalanga…………………………..<br>Kgalagadi…………………………<br>Herero……..……………………...<br>Bayeyi……………………………<br>Bambukush………………………<br>Basarwa…………………………<br>Other______________________<br>No response……………………	1<br>2<br>3<br>4<br>5<br>6<br>7<br>10<br>99
**Household headship**	AU9	**Who is the head of the household where you usually live?**<br>*Tlhogo ya lelwapa le o nnang mo go lone ke mang?*<br><br>**[ENTER ALL THAT APPLY.]**	Self……………………………......<br>Father…………………………. .. .<br>Mother……………..……………<br>Husband/Male Partner. ....……<br>Brother…………………………..<br>Eldest Son…………...…………..<br>Father-In-Law……………………<br>Mother-In-Law…………….........<br>Other………………………….. ..<br>________________________<br>(Specify)<br>No Response………..…………	11<br>12<br>13<br>14<br>15<br>16<br>17<br>20<br>21<br><br><br>99
	AU-HHG	**Gender/s of head of household based on response to AU11**	Female……………………………<br>Male ……………………………...<br>Shared (Male & Female)…………	0<br>1<br>2
**Marital status**	AU10	**What is your marital status now? Are you widowed, divorced, separated, or living together with a man as if married?**<br>*Seemo sa gago sa nyalo ke sefe? A o tlhokafaletswe ke monna, o tlhadile, o arogane le monna, kgotsa o nna le monna mo ntlong jaaka banyalani?*<br><br>**[Count as "married" anyone in process of marrying and where *patlo* has been paid.]**	Currently Married………. ………<br>Divorced or Separated …………...<br>Widowed…………………………<br>Living together……………………<br>Single……………………………<br>No Response……………………..	1<br>2<br>3<br>4<br>5<br>99
	AU11	**Number of times married/living with a man**<br>*Makgetlo a o nyetsweng/ o ntseng le monna mo ntlong.*<br><br>**[NOT boyfriends- only count times when respondent shared a household with a man]**	Married/Lived With A Man Only Once……………..........................<br>Married/Lived With A Man More Than Once………………………...<br>Not Applicable…………............<br>No Response……………………...	1<br><br>2<br>88<br>99
	AU12	**How old were you when you first got married or began to live with a man as in a union? [Share a household with a man?]**<br>*O ne o le digwaga di kae fa o nyalwa la ntlha kgotsa o simolola go nna le monna mo ntlong?*	Age At First Marriage Or Living together…………………………..<br>Doesn't know……………………<br>Not Applicable…………………..<br>No Response……………………..	__<br>11<br>88<br>99
**Husband/ Partner Characteristics**	AU13	**Age of partner at time of respondent's first marriage or union. [Shared household]**<br>*Dingwaga tsa monna fa nyalo e simologa / le simolola go nna mmogo.*	Age Of Partner …………………..<br>Doesn't know……………………<br>Not Applicable……………………<br>No Response……………………...	_ _<br>11<br>88<br>99
	AU14	**What is the highest level of school your CURRENT husband/partner attended: primary, secondary, or higher?**<br>*Sekolo se Sennye; Sekolo se Segolwane; Dithuto tse Dikgolo?*	No schooling……………………..<br>Primary ………………….........<br>Secondary………………………...<br>Higher …………………………<br>Highest standard/form/grade completed _______________<br>No husband/Partner……………..<br>No Response……………………..	*0*<br>*1*<br>*2*<br>*3*<br><br>*88*<br>*99*

		Questions and Filters	Coding Categories	Code
	AU15	**Does your husband/partner currently work for wages outside the home?** *A mo sebakeng seno monna/molekane wa gago o a bereka?*	Yes……………………………<br>No……………………………<br>Not Applicable **(NO PARTNER)**<br>No Response……………………	*1*<br>*0**<br>*88*<br>*99*
	AU16	***What kind of work does your husband do for wages?*** *Monna/molekane wa gago o dira tiro ya mofuta mang?*	*Agriculture………………………*<br>*Construction……………………..Dr*<br>*iver/Taxi………………………*<br>*Police/Mil………………………..*<br>*Bus/Adm………………………*<br>*Bus/Mgmt………………………..*<br>*Other…………………………..*<br>*Not applicable* ***(NO PARTNER)***<br>*No response………………………*	*1*<br>*2*<br>*3*<br>*4*<br>*5*<br>*6*<br>*7*<br>*88*<br>*99*

Now I would like to ask you some questions about children.
Jaanong ke batla go go botsa dipotso ka bana?

		Questions and Filters	Coding Categories	Code
**Children**	AU16	**How many living children do you have?** *O na le bana ba le kae ba ba mo botshelong?*	Number Of Living Children……….<br>Not Applicable…………….........<br>No Response ………………………<br><br>[*If No Living Children, Skip To AU18.]	_ _*<br>88<br>99
	AU17	**How old were you when your first child was born?** *O ne o le dingwaga di kae fa o tshola ngwana wa ntlha?*	Age At First Birth…………………..<br>Doesn't know………………………<br>Not Applicable……….……………..<br>No Response………………………..	__<br>11<br>88<br>99
	AU18	**Are any of your children under the age of 15 years**?	Yes………………………………..<br>If Yes, how many?_____________<br>No…………………………………<br>Not Applicable……………………...<br>No Response………………………..	1<br><br>0<br>88<br>99
	AU19	**Do these children (those under 15 years) live in your house with you?**	Yes………………………………<br>No………………………………….<br>If No, where do they live?<br>______________________________<br>Not applicable……………………….<br>No Response………………………...	1<br>0<br><br><br>88<br>99
	AU20	**Are you responsible for any children under the age of 15 years old who live with you who are not your own children?**<br><br>**[e.g. grandchildren, nieces, nephews, orphans of relatives]**	Yes………………………………<br>____________________________<br>Specify Whose Children<br>No………………………………….<br>Not Applicable…………………….<br>No Response……………………….	1<br><br><br>0<br>88<br>99

		Questions and Filters	Coding Categories	Code
	AU21	**Who in your household usually has the final say on the following decisions about the child(ren):** *Ke mmang yo gale a tsayang ditshwetso ka bana ba mo gae?*		
		**Any decisions about children's schooling?** *Dithulaganyo tsa go tsenya bana sekolo?*	Self……………………………........<br>Self w/husband/partner……………..<br>Husband/Partner alone……………<br>Self with other family members……<br>Other family members w/o self…….<br>Not Applicable……………….........<br>No Response………………………..	1<br>2<br>3<br>4<br>5<br>88<br>99
		**How children should be disciplined?** *Bana ba otlhaiwa jang?*	Self……………………………........<br>Self w/husband/partner……………..<br>Husband/Partner alone……………<br>Self with other family members……<br>Other family members w/o self…….<br>Not Applicable……………….........<br>No Response………………………..	1<br>2<br>3<br>4<br>5<br>88<br>99
		**Whether to have a child?** *Tshwetso ya go tshola ngwana?*	Self……………………………........<br>Self w/husband/partner……………..<br>Husband/Partner alone……………<br>Self with other family members……<br>Other family members w/o self…….<br>Not Applicable……………….........<br>No Response………………………..	1<br>2<br>3<br>4<br>5<br>88<br>99
		**Whether or not to use a method to avoid having children?** *Go thibela kgotsa go sa thibele pelegi?*	Self……………………………........<br>Self w/husband/partner……………..<br>Husband/Partner alone……………<br>Self with other family members……<br>Other family members w/o self…….<br>Not Applicable……………….........<br>No Response………………………..	1<br>2<br>3<br>4<br>5<br>88<br>99

		Questions and Filters	Coding Categories	Code
**Participation in healthcare decision-making as parent**	AU22	**Please tell me who in your household decides:** *Tswee tswee ntlhalosetsa gore mo gae ke mang yo o tsayang ditshwetso ka :*		
		**What to do when a child falls sick?** *Gore go dirwang fa ngwana a lwala?*	Self……………………………........<br>Self w/husband/partner……………..<br>Husband/Partner alone……………<br>Self with other family members……<br>Other family members w/o self…….<br>Not Applicable……………..........<br>No Response………………………..	1<br>2<br>3<br>4<br>5<br>88<br>99
		**When to take a child to the doctor or health clinic?** *Gore ngwana o isiwa ngakeng/ kokelwaneng (tliliniking) leng?*	Self……………………………........<br>Self w/husband/partner……………..<br>Husband/Partner alone……………<br>Self with other family members……<br>Other family members w/o self…….<br>Not Applicable……………..........<br>No Response………………………..	1<br>2<br>3<br>4<br>5<br>88<br>99
		**When to consult a traditional healer about the child?** *Gore ngwana o isiwa leng ngakeng ya setso?*	Self……………………………........<br>Self w/husband/partner……………..<br>Husband/Partner alone……………<br>Self with other family members……<br>Other family members w/o self…….<br>Not Applicable……………..........<br>No Response………………………..	1<br>2<br>3<br>4<br>5<br>88<br>99

Now I would like to ask you some questions about financial matters. I ask these questions only to understand more about the financial position of women in Botswana. Please remember that you do not have to answer any question that makes you feel uncomfortable. I can repeat any question that you do not understand.

Jaanong ke eletsa go go botsa dipotso mabapi le madi a go itshidisa. Ke botsa dipotsotse fela gore ke tlhaloganye sentle ka maemo a basadi ba Botswana a madi. Tsweetswee gakologelwa gape gore ga o patelesege go araba potso epe fela e e sa go tseng sentle. Ke ka boelela potso nngwe le nngwe e o sa e tlhaloganyang.

AU23	**Asset Independence**<br>**Please tell me if you alone, or jointly with (your husband or) someone else own:**<br>*Tswee tswee mpolelela gore a wena ka nosi o na le lefatshe, kgotsa o le tlhakanetse le monna wa gago, kgotsa le mongwe fela?*	N/R = NO RESPONSE<br>N/A = NOT APPLICABLE<br>DK = DOESN'T KNOW				**If you ever need to, can you sell (ASSET) without anyone else's permission?** *Fa go ka tlhokafala gore o rekise (Lefatshe/ Ntlo/ Meikgabiso/ loruo lo) a o ka dira jalo o sa tlhoke tetla ya ga ope?*				
		**DOES NOT OWN**	**OWNS JOINTLY**	**OWNS ALONE**	**N/R**	**YES**	**NO**	**DK**	**N/A**	**N/R**
	**Land?** *Lefatshe?*	1	2	3	99	1	2	3	88	99
	**The house/dwelling you live in?** *Ntlo/ bonno jo o nnang mo go jone?*	1	2	3	99	1	2	3	88	99
	**Livestock (cattle/ goats?)** *Loruo (dikgomo kgotsa dipodi)?*	1	2	3	99	1	2	3	88	99

		Questions and Filters	Coding Categories	Code
**Economic independence**	AU24	**Do you have any money of your own that you alone can decide how to use?**<br>*A o na le madi a gago o le nosi a o a laolang?*	Yes…………………………………..<br>No…………………………………...<br>No Response………………………..	1<br>0<br>99
	AU25	**Do you have a bank account or an account in any other savings institution in your own name or jointly with someone else?**<br>*A o na le polokelo ya banka kana polokelo nngwe fela e e mo leineng la gago kana e o e? tlhakanetseng le mongwe?*	Yes, In Own Name…………………<br>Yes, Joint Account…………………<br>No Account…………………………<br>No Response………………………<br><br>[*If No Account (3), Skip To AU26.]	1<br>2<br>3*<br>99
	AU26	**Do you yourself operate the account, that is, sign checks or deposit and withdraw money?**<br>*A ke wena o dirisang polokelo e, jaaka, go kwala ditsheke, go boloka le go tsaya madi?*	Yes…………………………………..<br>No…………………….…………..<br>Not Applicable……………………...<br>No Response………………………...	1<br>0<br>88<br>99
	AU27	**Do you know of any organizations that help women start their own businesses?**	Yes…………………………………<br>No………………………………..<br>No Response………………………	1<br>0<br>99
	AU28	**Have you yourself ever taken out or been given a loan either in cash or in kind to start or expand a business?** *A wena o kile wa adima madi kgotsa wa thusiwa go simolola kgotsa go atolosa kgwebo?*	Yes…………………………………..<br>No…………………………………...<br>No Response………………………..	1<br>0<br>99
	AU29	**If you need help or have a problem, is there someone from your family on whom you can depend to:**<br>*A wena o kile wa adima madi kgotsa wa thusiwa go simolola kgotsa go atolosa kgwebo:*<br><br>**Give you shelter for a few nights if you need it?**<br>*Go go fa boroko malatsinyana fa o bo tlhoka?*<br><br><br><br>**Give you financial support if you need it?**<br>*Go go thusa ka madi fa o a tlhoka?*	 <br><br><br><br><br>Yes…………………………………<br>No………………………………..<br>No Response………………………<br><br>Yes…………………………………<br>No………………………………..<br>No Response………………………	 <br><br><br><br><br>1<br>0<br>99<br><br>1<br>0<br>99
**Associational life**	AU30	**Are you part of a church, club, or group to which you go regularly?**	Yes…………………………………..<br>No…………………………………...<br>No Response………………………...	1<br>0<br>99
	AU31	**What kind of group or club is it?**<br>*Ke mokgatlho, lekoko, lekgotlana la mofuta mang?*<br><br>**[PROMPTS OKAY. RECORD ALL MENTIONED.]**	Religious………………………........<br>Social……………………………….<br>Women's Organization……………..<br>Labor Union………………………...<br>Political……………………………..<br>Other………………………………..<br>______________________________<br>(Specify)<br>Not Applicable……………..........<br>No Response………………………..	1<br>2<br>3<br>4<br>5<br>6<br><br><br>88<br>99
**Political participation**	AU32	**When there is a local or a national election of any kind do you vote always, sometimes, or never?** *Fa go na le ditlhopho tsa lefatshe kgotsa tsa kgaolo a o lthola o tlhopha, o tlhopha nako nngwe kgotsa ga o ke o tlhopha?*	Always Votes………………………<br>Sometimes Votes…………..........<br>Never Votes…………………………<br>Too Young To Vote………………...<br>No Response………………………..	1<br>2<br>0<br>88<br>99

		Questions and Filters	Coding Categories	Code
**Autonomy/ Household tasks and decisions**	AU33	**Please tell me who in your household decides the following:** *Tswee tswe mpolelela gore ke mang mo lapeng yo o o lalolang tse di latelang:*		
		**What food to buy for family meals?** *Gore go rekiwa dijo dife?*	Self…………………………….........<br>Self w/husband/partner……………..<br>Husband/Partner alone……………<br>Self with other family members……<br>Other family members w/o self…….<br>Not Applicable……………….........<br>No Response………………………..	1<br>2<br>3<br>4<br>5<br>88<br>99
		**Whether or not you should work outside your home for wages?** *Gore a o ka bereka kgotsa o ka nna fela mo gae?*	Self…………………………….........<br>Self w/husband/partner……………..<br>Husband/Partner alone……………<br>Self with other family members……<br>Other family members w/o self…….<br>Not Applicable……………….........<br>No Response………………………..	1<br>2<br>3<br>4<br>5<br>88<br>99
		**Whether or not to purchase major goods for the household such as a television or a radio?** *Gore a o ka reka dilwana tsa ntlo, jaaka redio/ seromamowa?*	Self…………………………….........<br>Self w/husband/partner……………..<br>Husband/Partner alone……………<br>Self with other family members……<br>Other family members w/o self…….<br>Not Applicable……………….........<br>No Response………………………..	1<br>2<br>3<br>4<br>5<br>88<br>99
	AU34	**When there is money in the household, can you decide on your own whether to buy any of the following?**		
		**Food?**	Yes………………………………….<br>No…………………………………...<br>Does Not Buy………………………<br>No Response……………………….	1<br>0<br>88<br>99
		**Clothes for yourself?** *Diaparo tsa gago?*	Yes………………………………….<br>No…………………………………...<br>Does Not Buy………………………<br>No Response……………………….	1<br>0<br>88<br>99
		**Any kind of medicine for yourself?** *Melemo epe fela e o ka e tlhokang?*	Yes………………………………….<br>No…………………………………...<br>Does Not Buy………………………<br>No Response……………………….	1<br>0<br>88<br>99
		**Toiletries for yourself (give a local example)?** *Melwara ya gago?*	Yes………………………………….<br>No…………………………………...<br>Does Not Buy………………………<br>No Response……………………….	1<br>0<br>88<br>99

		Questions and Filters	Coding Categories	Code
**Freedom of Movement**	AU35	**Do you need to ask permission to go to the following places:** *A go tlhokafala gore o ikope fa o a kwa mafelong a a latelang?*		
		**Any place outside your house or compound?** *Gope fela kwa ntle ga lelapa?*	Yes………………………………….<br>FROM WHOM_______________<br>No…………………………………..<br>No Response……………………….	1<br><br>0<br>99
		**The health center, clinic or doctor?** *Bookelo, tliliniki, ngakeng?*	Yes………………………………….<br>FROM WHOM_______________<br>No…………………………………..<br>No Response……………………….	1<br><br>0<br>99
		**The traditional healer?** *Ngakeng ya setswana?*	Yes………………………………….<br>FROM WHOM_______________<br>No…………………………………..<br>Not Applicable……………………..<br>No Response……………………….	1<br><br>0<br>88<br>99
		**To a nearby church or religious center?** *Kereke e e gaufi kgotsa kobamelo nngwe?*	Yes………………………………….<br>FROM WHOM_______________<br>No…………………………………..<br>Not Applicable……………………...<br>No Response……………………….	1<br><br>0<br>88<br>99
		**To the local market?** *Kwa mmarakeng?*	Yes………………………………….<br>FROM WHOM_______________<br>No…………………………………..<br>No Response……………………….	1<br><br>0<br>99
		**To homes of friends in the neighborhood?** *Malapa a ditsala tse di mabapi?*	Yes………………………………….<br>FROM WHOM_______________<br>No…………………………………..<br>No Response……………………….	1<br><br>0<br>99

AU36	**ATTITUDES TOWARD GENDER DOMAINS Please tell me if you agree or disagree with each statement:** *Tswee tswee supa gore a o a dumalana kgotsa ga o dumalane le ditemana tse di latelang:*	N/R = NO RESPONSE. DK = DOESN'T KNOW	**AGREE**	**DIS-AGREE**	**DK/ DEP-ENDS**	**N/R**
	**The important decisions in the family should be made only by the men of the family.** *Ditswhetso tsa botlhokwa di tshwanetswe go tsewa fela ke bana mo lapeng.*	FAMILY DECI-SIONS BY MEN	1	2	3	99
	**If the wife is working outside the home, then the husband should help her with household chores.** *Fa mosadi a bereka, monna ene o tshwanetse go mo thusa tiro ya lolwapa.*	HUSBAND SHOULD HELP	1	2	3	99
	**A married woman should be allowed to work outside the home if she wants to.** *Mosadi yo o nyetsweng o tshwanetse go letliwa go bereka fa a batla.*	WOMEN SHOULD WORK	1	2	3	99
	**The wife has a right to express her opinion even when she disagrees with what her husband is saying.** *Mosadi o na le tshwanelo ya go ntsha maikutlo a gagwe fa monna a bua se a sa dumalaneng le sone.*	WIFE TO EX-PRESS OPINION	1	2	3	99
	**A wife should tolerate being beaten by her husband in order to keep the family together.** *Mosadi o tshwanetse go itshokela go betswa ke monna fela gore atshegetse lelapa.*	TOLERATE BEING BEATEN	1	2	3	99
	**It is better to send a son to school than it is to send a daughter.** *Go botoka go tsenya ngwana wa mosimane sekolo go na le wa mosetsana.*	BETTER TO SCHOOL SON	1	2	3	99

Now I would like to ask you some questions about how familiar you are with medical clinics, hospitals, medical treatment. *Jaanong ke eletsa go go botsa dipotso ka kitso yya gago ka ditliliniki, dipatela, le kalafi kana bongaka.*

		Questions and Filters	Coding Categories	Code
**Familiarity with medical setting**	AU37	**Have you ever participated in research/ a clinical trial before?** *A o kile wa tsenelela ditekeletso tsa bongaka/ kalafi?*	Yes……………………………………. No…………………………………….. No Response…………………………..	1 0 99
	AU38	**Have you ever visited a medical clinic for:**		
		**Prenatal care?** *Tlhokomelo ya boimana?*	Yes……………………………………. No…………………………………….. Not Applicable……………………….. No Response…………………………..	1 0 88 99
		**Women's issues other than childbirth?** *Tsa sesadi fela tse di sa amaneng le bongaka?*	Yes……………………………………. No…………………………………….. Not Applicable……………………….. No Response…………………………..	1 0 88 99
	AU39	**How many times have you visited a medical clinic in the last year?**	# Of Times……………………………. Some times/A few times……………… Many Times………………………….. No Response………………………….	_ _ 66 77 99

	AU40	**How many times in the past twelve months have you visited a traditional healer?** *Mo selekanyong sa dikgwedi tse di lesome le bobedi, o ile ngakeng ya setswana ga kae?*	# Of Times……………………………<br>Some times/A few times………………<br>Many times……………………………<br>Not Applicable………………………..<br>No Response………………………….	_ _<br>66<br>77<br>88<br>99

		Questions and Filters	Categories	
**PRIME-MD Mood Module**	WH1	**<u>For the last two weeks</u>, have you had any of the following problems <u>nearly every day</u>?** *Mo bekeng tse pedi tse di fitileng a o kile wa nna mathata a a latelang letsatsi le negwe le negwe ka kakaretso?*		
	(1)	**Trouble falling or staying asleep, or sleeping too much?** *Go tlhorega/ ganwa ke boroko, kgotsa go robala thata?*	Yes………………<br>No………………..<br>No Response……..	1<br>0<br>99
	(2)	**Feeling tired or having little energy?** *Letsapa, kana go tlhoka moko?*	Yes………………<br>No………………..<br>No Response……..	1<br>0<br>99
	(3)	**Poor appetite or overeating?** *Go sulafalelwa ke dijo, kana go ja thata?*	Yes………………<br>No………………..<br>No Response……..	1<br>0<br>99
	(4)	**Little interest or pleasure in doing things?** *Go tlhoka kgatlhego mo go direng sepe?*	Yes………………<br>No………………..<br>No Response……..	1<br>0<br>99
	(5)	**Feeling down, depressed, or hopeless?** *Go ikutlwa o le kwa tlase, o ipona o se na mosola?*	Yes………………<br>No………………..<br>No Response……..	1<br>0<br>99
	(6)	**Feeling bad about yourself-or that you are a failure or have let yourself or your family down?** *Go ikutlwa o swabile, o itshulafalela – kgotsa o ikutlwa o paletswe ebile o digile bagalona seriti?*	Yes………………<br>No………………..<br>No Response……..	1<br>0<br>99
	(7)	**Trouble concentrating on things, such as reading the newspaper or watching television?** *Bothata jwa go nna le kelelelo, le o ka re o bala pampiri ya dikgang kana o lebile thelebishene tota?*	Yes………………<br>No………………..<br>No Response……..	1<br>0<br>99
	(8)	**[TWO-PART QUESTION]**<br>**Being so fidgety or restless that you were moving around a lot more than usual?** *Go babalelwa ke manno, o korakorega mo nnang ka go tsamaya tsamaya go feta gale?*<br><br>**If 'No', what about the opposite: moving or speaking so slowly that other people could have noticed?** *Mo gonngwe gone? – jaaka go tsamaya le go bua ka bonya mo batho ba ka tswang ba lemogile?* **[COUNT <u>YES</u> IF ANSWER IS YES TO EITHER QUESTION, OR IF PSYCHOMOTOR AGITATION OR RETARDATION OBSERVED DURING INTERVIEW.]**	Yes………………<br>No………………..<br>No Response……..	1<br>0<br>99
	(9)	**<u>In the last two weeks</u>, have you had thoughts that you would be better off dead or hurting yourself in some way? [If Yes] Tell me about it.** *Mo dibekeng tse pedi tse di fetileng a o kile wa iphitlhela o akanya gore go botoka o sule kana wa ikgobatsa ka mokgwa mongwe fela?* ***[If Yes]*** *Ntlhalosetse ka gone?*	Yes………………<br>No………………..<br>No Response……..	1<br>0<br>99

		Questions and Filters	Categories	
	AU-MDD	**<u>Results of Depression Screen:</u>**<br>If at least five of the above questions were answered yes, and AT LEAST ONE OF THE FIVE was question #4 or #5, CODE AU-MDD as "1"	MDD…………….<br>NOT MDD………<br><br>No Response……..	1<br>0<br><br>99

Now I would like to ask you some questions about the screening program that has just been described to you. Please ask me to explain any question that you do not understand.

		Questions and Filters	Coding Categories	Code
	IC1	**Did you understand everything that was explained to you about the screening program?** *A o tlhalogantse tsotlhe tse o di tlhaloseditsweng mabapi le lenaneo la tlhatlhobo?*	YES……………………………….<br>NO…………………………………<br>NO RESPONSE…………………...	1<br>0<br>99
**Understanding**	IC2	**Can you tell me the purpose of the screening program?** *A o ka ntlhalosetsa lebaka la lenaneo le la tlhatlhobo?*<br><br><u>EXPLANATION GIVEN</u>:	YES, APPROXIMATE………..<br>CANNOT TELL PURPOSE………<br>NO RESPONSE…………………	1<br>0<br>99
	IC3	**Do you know what cancer of the cervix or cervical cancer is?** *A o itse gore kankere ya molomo wa popelo ke eng?*	YES……………………………….<br>NO……………………………….<br>NO REPONSE……………………	1<br>0<br>99
	IC4	**Can you tell me what causes cervical cancer?**<br><br><u>EXPLANATION GIVEN</u>:	YES,<br>APPROXIMATE…………………..<br>CANNOT TELL CAUSE…………<br>NO RESPONSE…………………..	 1<br>0<br>99
	IC5	**Do you think HIV lowers a woman's chances of getting cervical cancer, raises her chances, or has no effect at all?** *O akanya gore HIV (mogare) e ka rotloetsa jang le gore mosadi a tsenwe ke kankere ya popelo?*	LOWERS HER CHANCE………<br>NO EFFECT……………………..<br>RAISES HER CHANCE…………<br>DOESN'T KNOW………………..<br>NO RESPONSE…………………	1<br>2<br>3<br>0<br>99
	IC6	**Can you tell me what HPV or human papillomavirus is?** *A o itse gore mogare wa kankere (HPV) ke eng?*<br><br><u>EXPLANATION GIVEN:</u>:	YES………………………………..<br>NO…………………………………<br>NO RESPONSE………………….	1<br>0<br>99

		Questions and Filters	Coding Categories	Code
	IC7	**Are you more likely, as likely or less likely to get [H PV] if you have HIV?**<br><br>[Instead of [HPV] use the respondent's own definition of HPV from above, e.g. this 'virus seed']	CORRECT ANSWER………………<br>WRONG ANSWER……..…………<br>NO RESPONSE…………………..	1<br>0<br>99
	IC8	**Who is going to do the screening for you?** *Ke mang yo o tlileng go go dira tlhatlhobo e?*<br><br>[DO NOT PROMPT]	A NURSE………………………..<br>A DOCTOR………………………<br>DOESN'T KNOW…………….…<br>NO RESPONSE…………….………	1<br>2<br>0<br>99
	IC9a	**If there are no problems with your screening today, when should you come back next for another exam?**	IN ONE YEAR……………………<br>WHEN______________________<br>[if other than 1 year]<br>DOESN'T KNOW………………...<br>NO RESPONSE…………………...	1<br>0<br><br>3<br>99
	IC9b	**If there are problems found with your screening today, when should you come back for a follow-up exam?**	IN SIX MONTHS………………….<br>WHEN ______________________<br>[if other than 6 mos]<br>DOESN'T KNOW………………….<br>NO RESPONSE…………………….	1<br>0<br><br>3<br>99
	IC10	**Will you see a doctor today?** *A o tlile go bona ngaka gompieno?*	YES…………………………………<br>NO………………………………….<br>DOESN'T KNOW………………....<br>NO RESPONSE……………….......	0<br>1<br>3<br>99
	IC11	**Will the person doing the exam use a camera?**<br><br>[If 'NO' or 'DOESN'T KNOW', skip to IC14]	YES…………………………………<br>NO………………………………….<br>DOESN'T KNOW………………....<br>NO RESPONSE……………….......	1<br>0<br>3<br>99
	IC12	**What will this person take a picture of?**	CERVIX/'INSIDES'………………..<br>INCORRECT ANSWER…………...<br>SPECIFY___________________<br>DOESN'T KNOW………………….<br>NO RESPONSE…………………….	1<br>0<br><br>3<br>99
	IC13	**Can you tell me how the picture will be used?**<br>*A o ka mpolelela gore setshwantsho se ya go dirisiwa jang?*	TO DETECT PROBLEMS………<br>OTHER (incorrect) REASON<br>___________________________<br>DOESN'T KNOW………………..<br>NO RESPONSE…………………	1<br><br>0<br>3<br>99
	IC14	**Could you be injured as a result of participating in the screening?** *A o ka gobadiwa ke go dira tlhatlhobo e?*	NO……………………………..<br>YES………………………………<br>DOESN'T KNOW………………<br>NO RESPONSE…………………	1<br>0<br>3<br>99
	IC15	**Did you understand enough about the screening program to be able to make your own decision to participate or not to participate?** *A o tlhalogantse sentle ka tlhatlhobo e mo o ka fetsang mogopolo gore o tswelela ka yone kgotsa o a e lesa?*	NOT ENOUGH……………………<br>UNDERSTOOD SOMEWHAT…..<br>UNDERSTOOD ENOUGH……….<br>UNDERSTOOD EVERYTHING…<br>NO RESPONSE…………………	0<br>1<br>2<br>3<br>99
**Voluntariness/ As a component of understanding**	IC16	**Did you have a choice whether or not to take part in the screening program?** *A o ne o ithaopa kana o patelediwa go tsenelela tlhatlhobo e?*	YES……………………………..<br>NO……………….……………..<br>DOESN'T KNOW………………<br>NO RESPONSE…………………...	1<br>0<br>3<br>99
	IC17	**Can you change your mind about being part of the screening program?** *A o ka fetola mogopolo mabapi le go tsenelela lenaneo la tlhatlhobo?*	YES………………………….…..<br>NO…………………………………<br>DOESN'T KNOW…….…………..<br>NO RESPONSE…………………...	1<br>0<br>3<br>99

		Questions and Filters	Coding Categories	Code
**Voluntariness/ As an action choice**	IC18	**Did you feel any pressure from anyone to agree to be in the screening program?** *A o ne o gatelelwa ke mongwe gore o dumele go tsenelela lenaneo la tlhatlhobo?*<br><br>**What is your relationship to this person?** *O amana jang le motho yoo?*	NO……………………………….<br>YES……………………………<br><br>WHAT IS YOUR RELATIONSHIP TO THIS INDIVIDUAL?<br>___________________________<br>(SPECIFY)<br>NO RESPONSE…………………	1<br>0<br><br><br><br><br><br>99
	IC19	**Did you have to consult with anyone before deciding about the screening program?** *A o ne o tshwanelwa ke go buisana le mongwe pele ga o dira tshwetso ka tlhatlhobo e?*<br><br>**With whom do you need to consult before making a decision?** *Ke mang yo o neng o tshwanela go buaisana le ene pele ga o dira tshwetso?*	NO……………………………….<br>YES………………………………<br><br>WHAT IS YOUR RELATIONSHIP TO THIS INDIVIDUAL?<br>___________________________<br>(SPECIFY)<br>NO RESPONSE………………….	1<br>0<br><br><br><br><br><br>99
	IC20	**Did you need anyone's permission in order to agree to be in the screening program?** *A o tlhoka tetla ya mongwe gore o fetse mogopolo ka go dira tlhatlhobo?*<br><br>**Whose permission did you need?** *O ne o tlhoka tetla ya ga mang?*	NO…………………………………<br>YES………………………………..<br><br>WHAT IS YOUR RELATIONSHIP TO THIS INDIVIDUAL?<br>___________________________<br>(SPECIFY)<br>NO RESPONSE…………………	1<br>0<br><br><br><br><br><br>99
	IC21	**Do you come to Bontleng Clinic for reasons other than this screening?**	YES…………………………………<br>NO………………………………….<br>NO RESPONSE……………………<br><br>[IF 'YES' Continue to IC22; if 'NO', skip to IC23.]	1<br>0<br>99
	IC22	[Only ask this question if woman comes to Bontleng Clinic for reasons besides screening.]<br><br>**If you refuse to be in the screening program, will this affect the OTHER care you receive here at the clinic?** *Fa o ka gana go tsenelela tlhatlhobo e, a se se ka kgoreletsa thuso/ kalafi e o ka e tlhokang mo tliliniking e?*<br><br>**In what way?** *Ka tsela efe?*	NO……………………………….<br>YES………………………………..<br>IN WHAT WAY?<br>___________________________<br>(SPECIFY)<br>DOESN'T KNOW……………….<br>NO RESPONSE…………………	1<br>0<br><br><br><br>3<br>99
	IC23	[Only ask this question if woman came to Bontleng only because she was referred for screening.]<br><br>**If you refuse to be in the screening program today, could you change your mind later and come back for a screening?**	YES…………………………………<br>NO………………………………….<br>DOESN'T KNOW…………………<br>NO RESPONSE……………………	1<br>0<br>3<br>99

Thank you very much for your time. *Ke lebogela nako ya gago thata.*

Number of people with participant at time of interview ______ Male/s

Relationship/s, if known: __________________________

______ Female/s

Relationship/s, if known: __________________________

Enter end time on first page of interview instrument.
